# 2-Dimensional (2D) Beam Steering-Antenna Using Active PRS for 5G Applications

**DOI:** 10.3390/mi14010110

**Published:** 2022-12-30

**Authors:** Misha Nadeem, Nosherwan Shoaib, Aimen Raza, Warda Saeed, Imran Shoaib, Sultan Shoaib

**Affiliations:** 1 School of Electrical Engineering and Computer Science (SEECS), National University of Sciences and Technology (NUST), Islamabad 44000, Pakistan; 2 Department of Electrical Engineering, Institute of Space Technology (IST), Islamabad 44000, Pakistan; 3 School of Electronic Engineering and Computer Science, Queen Mary University of London, London E1 4NS, UK; 4 Communication and Industrial Engineering, Faculty of Arts, Science and Technology, Department of Engineering, Wrexham Glyndwr University, Wales LL11 2AW, UK

**Keywords:** Fabry-Perot-cavity, partial reflection, microstrip patch antenna, beam-steering, microwave frequency band

## Abstract

This paper presents a compact coaxial-fed square microstrip patch antenna integrated with a beam-steering Partially Reflective Surface (PRS). The proposed design has a two-dimensional (2-D) Fabry-Perot Cavity (FPC) antenna acting as a radiator and the PRS as a beam-steering superstrate operating at 5.5 GHz. The PRS consists of 6 × 6 reconfigurable unit cells etched on Rogers 5880 with a thickness of 1.57 mm. By controlling the switching of PIN diodes in different sections of PRS, beam steering can be achieved up to ±47° in the azimuth plane with a gain of 9.1 dB and 10.2 dB and ± 15° steering in an elevation plane with a measured gain of 9.3 dB and 8.9 dB, respectively. The antenna prototype is 2.56 λ × 2.56 λ at 5.5 GHz, and the measured gain values are around 10 dBi for all the states. The beam is able to radiate at boresight or tilted to 42° in two azimuthal directions and 45° in elevation direction by adjusting the PIN diodes ON/OFF states. The 10 dB impedance bandwidth of 5.4–5.52 GHz is achieved.

## 1. Introduction

Frequency Selective Surface (FSS) has drawn a lot of attention owing to its merits of the broadside pattern, low loss and cost, ease of fabrication, and high degree of integration. The FSS is composed of a periodic structure having 2-Dimensional (2D) arrays of unit cells. Depending on the arrangement of the unit cells, it causes the incoming electromagnetic (EM) wave to either transmit or reflect, partially or completely, in a particular direction. In literature, novel designs have been presented for applications such as polarization converters [1], beam deflectors [2], and flat lenses [3]. The FSS used in [4] behaves as a reflector as it fully reflects the incident wave which results in an increase in the gain of the antenna. In [5], a wide band circular polarization antenna is designed with a partially reflecting surface. In-between the antenna and partially reflecting surface, multiple reflection phenomenon occurs, and incoming wave transmits in a specific direction, and the gain of the antenna increases. Similarly, a single layer reflector FSS is used in [6] with an omni-directional antenna to increase the gain.

The electronically tunable FSS, also known as reconfigurable FSS, has been explored more lately. By embedding different active elements like varactor diodes, MEMS switches, and PIN diodes reconfigurable FSS can realize different tunable responses like frequency adjustment [7], tunable absorber [8], and beam-steering [9]. The reconfigurable FSS are of two types; one is fully reflected/transmitted. When the diodes are in the ON state, it acts as fully reflected surface and when all the diodes are in the OFF state, it acts as fully transmitted or vice versa depending on the nature of the FSS. This type of FSS is used with a dipole or omni-directional antennas to achieve the beam steering in 360°. It is necessary to make the radiation pattern directive using such reconfigurable FSS to reduce interference to nearby systems. For this purpose, 3-Dimensional (3D) structures of fully reflected/transmitted active FSS (AFSS) are available. A cylindrical shape tunable FSS design is proposed in [8,9] for dual band beam swiping of a dipole antenna. The electronic beam steering is achieved in [10,11,12,13,14,15] by employing hexagonal/cylindrical AFSS structures. To achieve compactness in size, substrate-based antennas are preferred, whereas to achieve the beam steering capabilities, reconfigurable partially reflective surfaces (PRSs) are employed.

The PRS antenna is categorized under a center-fed leaky-wave antenna with a high gain and low profile. It is used to generate a broadside or tilted beam. It is comprised of a micro-strip patch antenna as a radiating source and a periodic or non-periodic array of geometrical patches or apertures made of the dielectric or metallic sheet as the PRS is located at the top of the source. The coaxial feed patch antennas are preferred in new designs due to their boresight radiation pattern and their compactness with the center feeding technique [15]. To enhance the directivity of an antenna at resonance, the distance between the source antenna and PRS should be a minimum of approximately a quarter of the wavelength.

The beam steering is achieved by placing a PRS over the antenna element to realize a varying reflection phase distribution. A PRS antenna has been presented with independent beam-scanning and beamwidth control capability. The beamwidth is controlled through the embedded varactor diodes while beam steering is achieved by using a small, phased array in the Fabry-Perot cavity. The achieved beam width varies from 18.7° to 22.4° with a scanning range of 15° and 20° [16]. A reconfigurable PRS antenna has been presented which provides a beam steering from −15° to +15°, an overlapped frequency ranges from 5.5 to 5.7 GHz, and a measured gain of around 12 dBi [17]. A dual-polarized FPA with PRS is proposed using an aperture-couple patch antenna which exhibits two different linear polarizations. The design provided a 10° beam tilt with respect to the broadside direction in eight different azimuthal directions. However, the design has employed 144 PIN diodes in the PRS which makes it a relatively lossy design [18]. A 2-D beam-steering PRS antenna was proposed by employing a reconfigurable PRS which can steer beam up to 22° in eight azimuthal directions with a less gain drop [19]. A 1-D PRS has been proposed to achieve a large beam steering range with a small gain variation using PRS superstrate. By using large trapezoidal patches, a high phase difference between different states of the unit cell exists which eventually leads to a large beam steering angle. The measured beam steering angle is ±18° with an operating frequency band of 5.45–5.65 GHz and a measured gain of over 11 dBi [20].

This paper proposed a beam-steerable PRS antenna with an improved angle and radiation performance. The reconfigurable PRS is designed using 6 × 6 reconfigurable unit cells divided into four equal sections having 3 × 3 unit cells each. The design of the unit cell consists of two half-square rings with two PIN diodes placed between the two gaps. The proposed antenna radiates a boresight beam in the OFF state and can steer the beam to around ±45° for *ϕ* = 0° and 90° for other diode states. By changing the PIN diodes states of PRS unit cells, the beam steering angle up to 46° is realized which is higher than [16]. Moreover, the proposed design only utilized 72 diodes which is only one-third used in [17] and half of that in [18]. The proposed design has achieved the 10-dB impedance bandwidth from 5.4 to 5.52 GHz with a realized gain of approximately 10 dBi. The paper organization is stated as follows: Section 2 highlights the design and analysis of the proposed unit cell. The results are highlighted in Section 3 followed by the conclusion in Section 4.

## 2. Design Methodology

**Figure 1:**
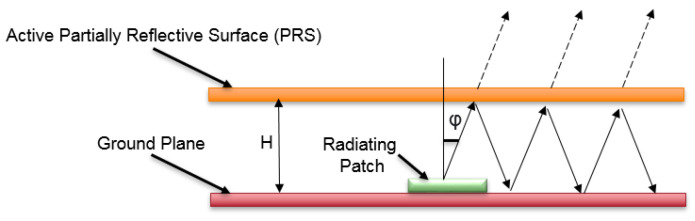
The active PRS structure conceptual diagram.

The proposed beam-steerable PRS structure is designed at 5.5 GHz for beam switching purposes. The beam-steerable PRS structure consists of two parts: a directional patch antenna with coaxial feed which acts as a radiating element and a reconfigurable active PRS which is placed at a certain height above the patch antenna. By placing the active PRS over the radiating patch antenna, a Fabry–Pérot cavity is formed as shown in Figure 1. The radiations from the patch antenna experience multiple partial reflections and transmissions depending on the impedance provided by the active PRS. The passband and stopband filtering operation of the active PRS is controlled by the ON/OFF states of PIN diodes. The radiating beam tilts in the direction where it experiences low reflections.

- Antenna Design

**Figure 2:**
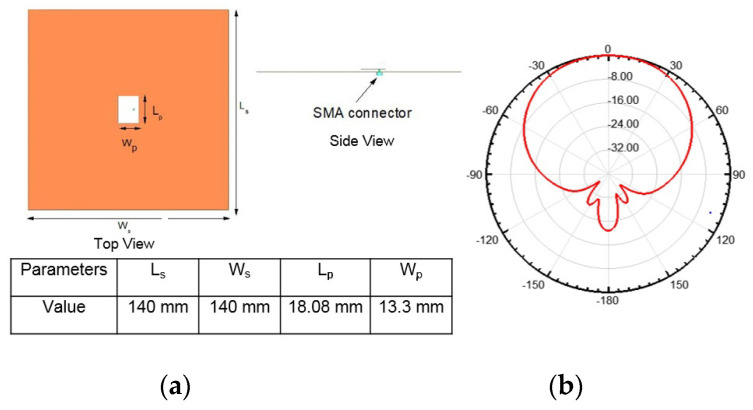
(**a**) Coaxial feed patch antenna (**b**) Boresight rediation pattern.

A coaxial feed microstrip patch antenna is designed at Rogers RT4003 substrate (with a thickness of 1.52 mm), which acts as the source antenna at 5.5 GHz. Its length and width are calculated using the mathematical formulations for a patch antenna (see Equations (1)–(3)) [21]. The radiating patch has dimensions of 13.3 × 18.08 mm^2^ at 5.5 GHz frequency. It has a boresight radiation pattern with a gain of 6.2 dBi. The antenna dimensions and radiation pattern are shown in Figure 2.

(1) $$Width=c/(2fo\left(\surd \left(\frac{\epsilon r+1}{2}\right)\right)$$

(2) $$\epsilon eff=\frac{\epsilon r+1}{2}+\left(\frac{\epsilon r-1}{2}\right)\left[\frac{1}{\sqrt{1+12\left(\frac{h}{w}\right)}}\right]$$

(3) $$Length=\frac{c}{2fo\sqrt{\epsilon eff}}-0.824h(\frac{\left(\epsilon eff+0.3\right)\left(\frac{w}{h}+0.246\right)}{\left(\epsilon eff-0.258\right)\left(\frac{w}{h}+0.8\right)}$$

II. Proposed Unit Cell

**Figure 3:**
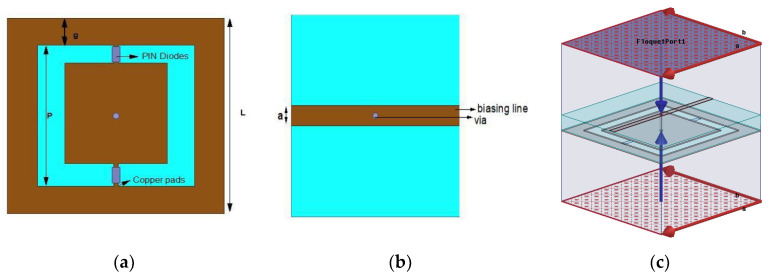
The conceptual design of unit cell (**a**) Front-view and (**b**) Back-view (**c**) Unit cell with view with Floquet Ports.

The conceptual design of the proposed reconfigurable unit cell is shown in Figure 3. It consists of a square looped patch enclosed in a mesh grid etched on Rogers 4003 with a thickness of 1.57 mm. The performance of the proposed unit cell has been verified by using periodic conditions in CST Studio Suite^®^. The Infineon BAR64 series diodes (BAR6402VH6327XTSA1) have been used in this design as they provide an isolation of 15 dB and less than 0.3 dB insertion loss at the resonant frequency.

**Figure 4:**
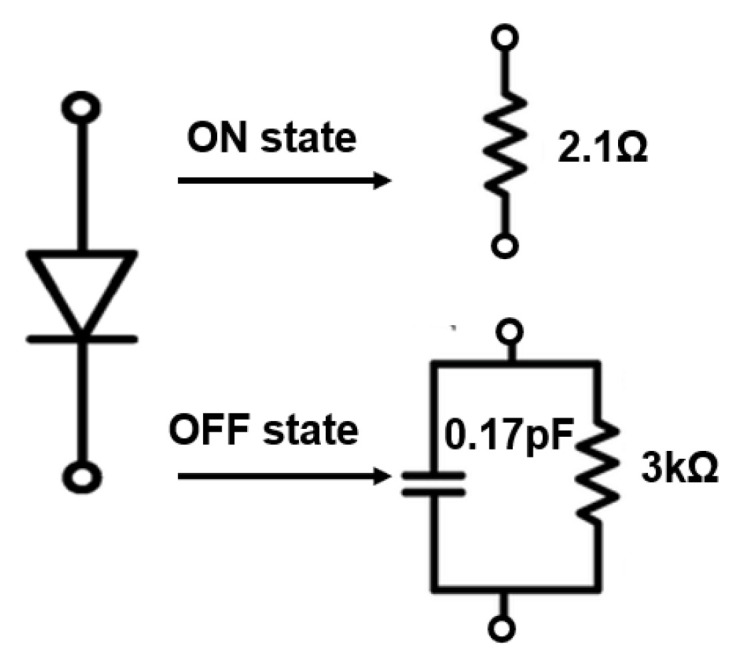
PIN diode equivalent circuit model.

Two PIN Diodes are placed between a patch and an outer metallic ring as shown in Figure 3a. The metallic strip is incorporated at the bottom side of a unit cell to provide biasing to the structure. The outer metallic ring is used for grounding purposes. The unit cell is excited with two Floquet ports shown in Figure 3c, Master-slave boundaries are used to model the periodic structure of the unit cell in the *x* and *y* direction. The PIN diodes are modeled as a lumped element circuit having a resistance of 2.1 Ω for the ON state, while for the OFF state, a parallel circuit composed of 0.17 pF capacitor and 3 kΩ resistor is employed as shown in Figure 4. The dimensions of the proposed unit cell are given in Table 1. All the parameters are chosen in such a way that when a PIN Diode changes its state from ON to OFF, there is a change in reflection magnitude & transmission phase.

**Figure 5:**
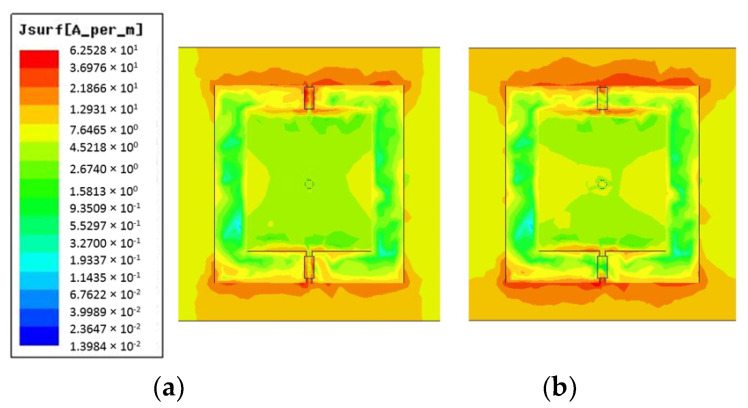
The surface current distribution analysis of the unit cell in (**a**) ON state (**b**) OFF state.

**Figure 6:**
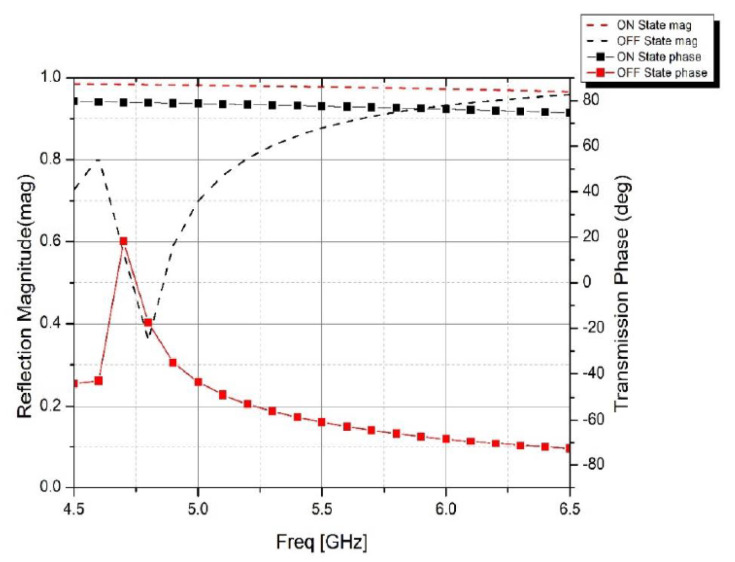
The reflection coefficient magnitude and the transmission coefficient phase results of the PRS unit cell.

Figure 5 shows a comparison of surface current distributions at ON and OFF states. The majority of the current passes through the middle diode and up to the end of the middle bar. It is then symmetrically divided and runs along the boundaries of the patch. The magnetic field forms a loop around the middle bar in response to the surface current distribution. The EM energy is stored in the loop, which prevents the plane wave from going through. When the top layer is in transmission mode, the surface current on the biasing network decreases dramatically, indicating that the magnetic loop trap becomes weak, and the EM wave can pass through. The reflection coefficient and transmission phase of the proposed unit cell is illustrated in Figure 6. It is evident that the reflection magnitude for the OFF- and ON-state are 0.87 and 0.97, respectively. The transmission phase varies from 77.5° in the ON state to −60.86° in the OFF state which exhibits a phase difference of 16.7°.

**Figure 7:**
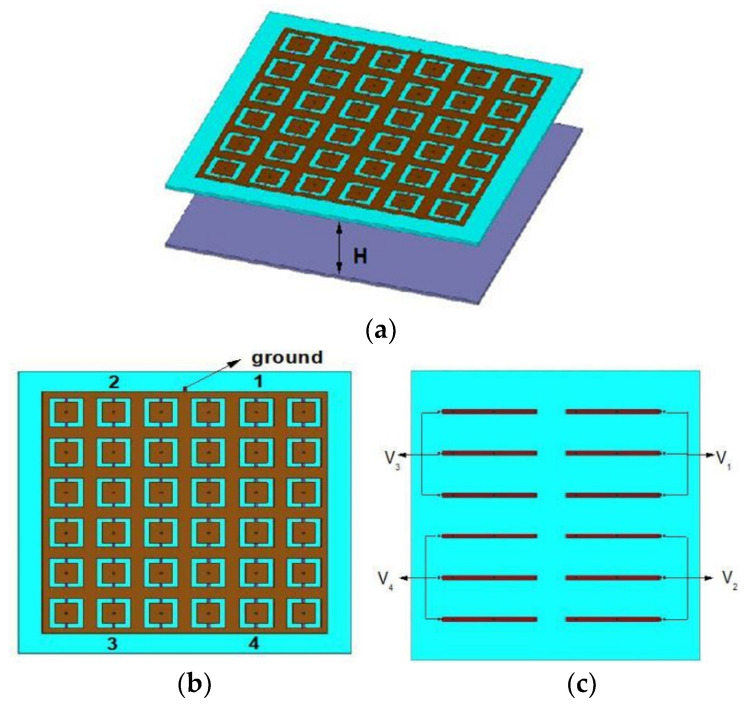
(**a**) Overall PRS structure having PRS placed at a height “H” from the antenna (**b**) The top-view of active partially reflective surface (PRS) (**c**) The bottom-view of the active partially reflective surface (PRS).

Following the working principle of a phased array antenna source, the antenna beam is shifted toward that side where the phase lags [10]. The reconfigurable FSS model is divided into four parts with each part consisting of 6 × 6 unit cells as shown in Figure 7. Each section has 3 × 3 reconfigurable unit cells. A total of 72 PIN diodes are used in the AFSS. An identical biasing network is designed for all four sections. It consists of thin metallic biasing strip-lines and biasing pads for voltage inputs, which can be seen from Figure 7 named sequentially as V_1_ to V_4_. The biasing strip lines are used to connect the cells, in each column, in series. The EM waves emitting out from the antenna undergoes multiple reflections within the cavity formed. A center-fed micro-strip patch antenna exhibits a broadside radiation pattern with a uniform PRS and a maximum broadside gain is achieved when the height between PRS and antenna is given by ray theory [4] and the mathematical formulation is mentioned as follows:

(4) $$H=\left(\frac{\varphi }{\pi \;}-1\right)\frac{\lambda }{4}+N\;\frac{\lambda }{2}\;,\;N=0,1,2..$$

where, *H* denotes the distance between PRS and antenna (i.e., cavity height)*, λ* is the wavelength which is calculated at the center frequency, and *φ* denotes the reflection phase of PRS in ON State, which is equal to 163° at 5.5 GHz, and N is the resonance order. Here *N* is considered as zero, i.e., the resonance of zeroth order. According to (1), *H* is calculated to be 27.04 mm, which is later optimized to 27.7 mm. Table 2 summarizes the state information and their relevant radiation directions. The detailed PRS performance analysis is mentioned in the next section.

## 3. Results and Discussions

**Figure 8:**
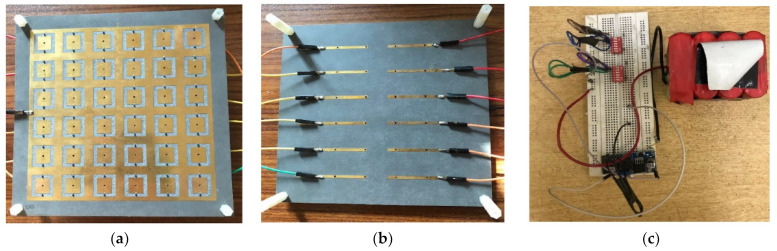
Fabricated Structure along with Biasing Network (**a**) Top-view (**b**) Bottom-view (**c**) DC Biasing Circuitry.

The proposed design is fabricated, and its performance is measured and compared with the simulated design results. Initially, a patch antenna is fabricated, and for simplicity, a planar FSS was fabricated. After that, a cavity is designed using Nylon spacers of 2.9 mm. The height between PRS and antenna is kept precisely to 27.7 mm, which is measured using a Vernier caliper. The overall structure has a size of 140 × 140 mm^2^. For biasing this structure, a separate circuitry is designed in the last step. A 12 V power supply is connected to the voltage regulator to regulate it to the desired value of 1.2 V. Two switches are used to control the four sections on the FSS structure, and four 18 Ω resistors are used to limit the current, as shown in Figure 8.

**Figure 9:**
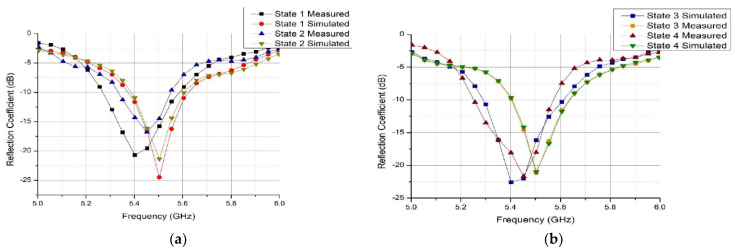
Reflection coefficient results of the proposed structure (**a**) States 1 and 2 (**b**) States 3 and 4.

The reflection coefficient measurements of the antenna are conducted using Anritsu MS46122B Agilent Vector Network Analyzer [22]. It can be seen from Figure 9a that the 10-dB bandwidth of the simulated reflection coefficients of state 1 and state 2 are 5.29 to 5.6 GHz and 5.292 to 5.6 GHz and measured reflection coefficients are 5.24 to 5.6 GHz and 5.26 to 5.65 GHz, respectively. Moreover, Figure 9b shows that the 10-dB bandwidth of the simulated reflection coefficient of state 3 and state 4 are 5.4 to 5.62 GHz and 5.4 to 5.62 GHz, and measured reflection coefficients are 5.23 to 5.58 GHz and 5.25 to 5.6 GHz, respectively. The minor difference between the measured and simulated results are due parasitic effects of the actual PIN diodes. However, the four states agreed to each other with an achieved 10-dB impedance between 5.24 to 5.6 GHz frequency band.

**Figure 10:**
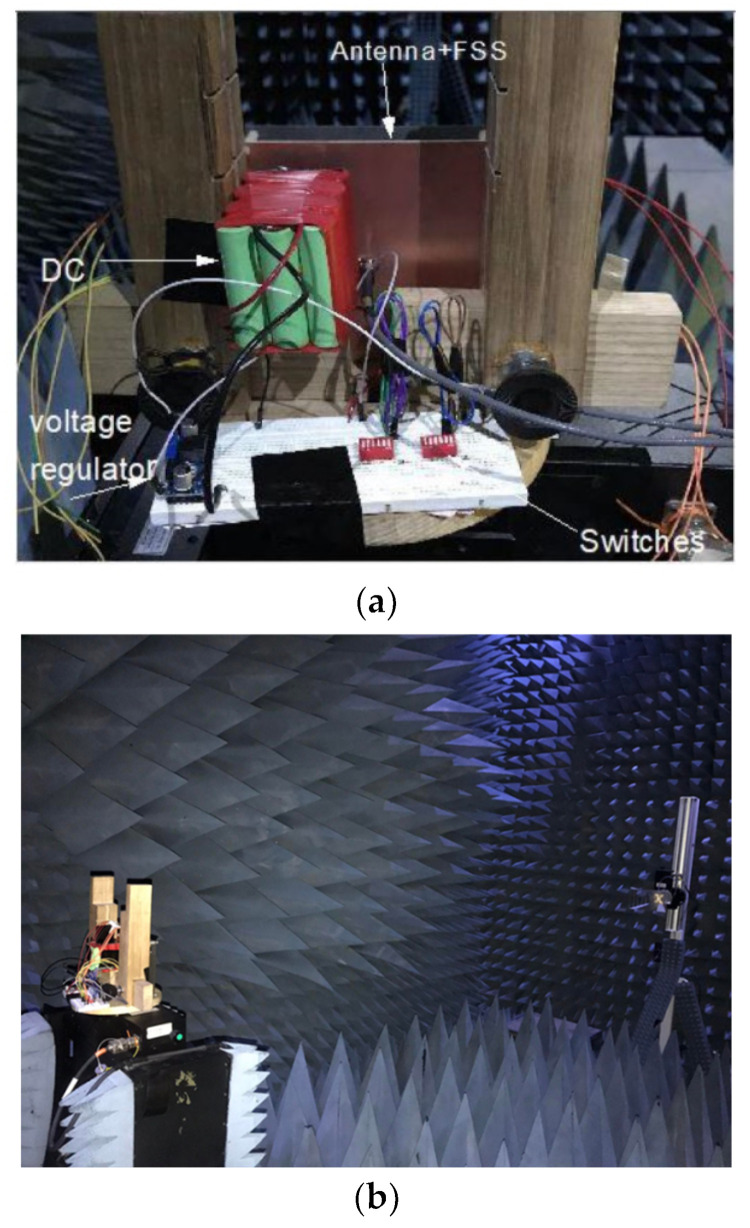
Far Field measurement of the fabricated prototype (**a**) Far-field setup (**b**) Strcuture Placement and Biasing.

**Figure 11:**
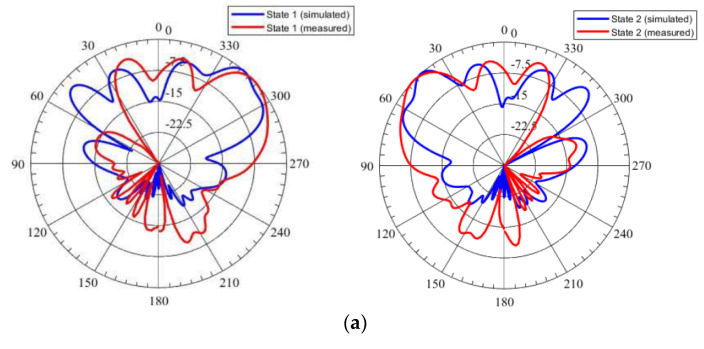

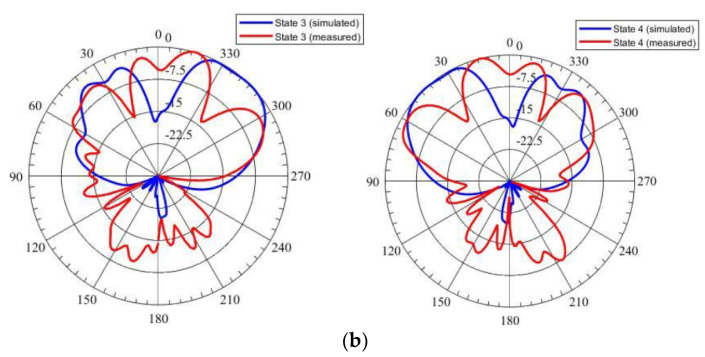
The radiation patterns result at 5.5 GHz (**a**) φ = 90° (**b**) φ = 0°.

The gain and radiation patterns measurements were carried out using the anechoic chamber facility available at NUST H-12 Campus, Islamabad, Pakistan. The proposed PRS antenna was placed at a far-field distance of 3.4 m from the transmitting wideband horn antenna whose operating frequency range is from 0.8 GHz to 18 GHz as shown in Figure 10. The radiation patterns for states 1, 2, 3, and 4 are shown in Figure 11. The simulated & measured results seem to be in good agreement in the Azimuth plane. For states 1 and 2, the beam steered at an angle of 46° and −45° from the broadside in the plane of *φ* = 90° with measured gains of 9.1 dBi and 10.2 dBi respectively. For States 3 and 4, the beam steered at an angle of 17° and −15° in the plane of *φ* = 0° with measured gains of 8.9 dBi and 9.3 dBi respectively. It was investigated that difference in beam tilt in the plane of *φ* = 0° (i.e., elevation plane) is due to the tolerances in the height of the PRS as the proposed design is sensitive to the height of the PRS. A slight inaccuracy in height between AFSS and antenna causes a large difference in beam tilt in the elevation plane. This aspect will be further investigated in future work and fabrication tolerances to maintain the height between AFSS and antenna will be minimized.

**Figure 12:**
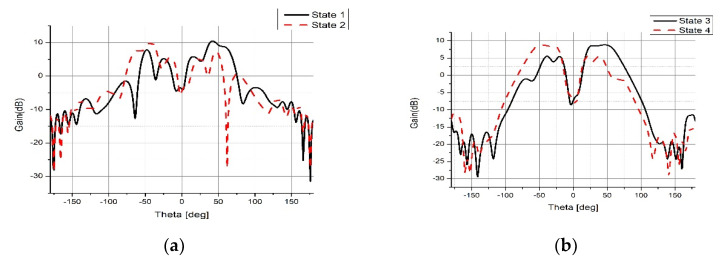
Gain Patterns (**a**) For Azimuth Plane (**b**) For Elevation Plane.

**Figure 13:**
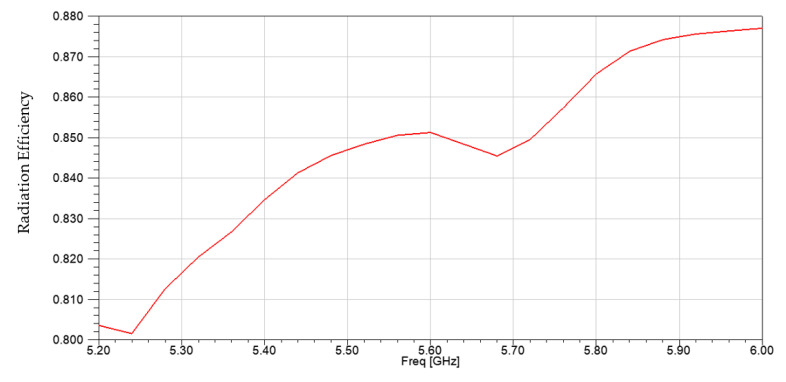
Radiation efficiency values.

The gain pattern for all states are shown in Figure 12. The radiation efficiency results are shown in Figure 13 where an efficiency of greater than 84% is achieved at 5.5 GHz. The simulated and measured gain values for all four states at 5.5 GHz are shown in Table 3 which agree with each other very well. Table 4 provides a performance comparison of the proposed design with the state-of-the-art. In comparison with the design proposed in [18,20], the proposed design is able to realize a much wider beam tilt; with a much smaller size compared to [18]. The beam tilt range of the proposed design is almost double as compared to [23,24] with a reduced antenna size.

## 4. Conclusions

A 2-D beam steering reconfigurable PRS-based antenna with an improved radiation performance is presented in this paper. The reconfigurable FSS model is divided into four parts with each part consisting of 6 × 6 unit reconfigurable cells placed at a height over a micro-strip patch antenna which acts as a radiating source. A total of 72 PIN diodes are used. By changing the states of the PIN diodes, the antenna is able to provide a maximum beam tilt of up to 46° with an impedance bandwidth of 5.4 to 5.52 GHz. For all states, the obtained realized gains are approximately 10 ± 1 dBi. A radiation efficiency of greater than 84% is achieved at 5.5 GHz. However, further optimization of this design could result in a lower side lobe level and a greater number of beam steering steps. The proposed FSS-based antenna structure can be a suitable candidate for future 5G wireless communication applications.

## Figures and Tables

**Table 1:** Dimensions of the proposed unit cell (in mm).

Parameters	L	P	G	a
Value	20	14.5	2.25	2

**Table 2:** States and Beam Tilt of the Proposed Antenna.

State	Sec-01	Sec-02	Sec-03	Sec-04	Beam Direction
1	ON	OFF	OFF	ON	φ/θ = 90°/42° (Azimuth Plane)
2	OFF	ON	ON	OFF	φ/θ = 90°/−42° (Azimuth Plane)
3	ON	ON	OFF	OFF	φ/θ = 0°/46° (Elevation Plane)
4	OFF	OFF	ON	ON	φ/θ = 0°/−45° (Elevation Plane)
5	OFF	OFF	OFF	OFF	Broadside

**Table 3:** Gain values for different states.

State	Simulated Gain	Measured Gain
**1**	10.5 dB	9.1 dB
**2**	10.2 dB	10.2 dB
**3**	8.83 dB	8.8 dB
**4**	8.81 dB	8.7 dB

**Table 4:** Comparison with the state-of-the-art.

Ref #	[18]	[20]	[23]	[24]	This Work
**Beam Steering**	±15°	±10°	±22°	±18°	±45° (Azimuth)
**Gain**	5–16.1 dBi	8.7–9.7 dBi	9.3–10.4 dBi	11 ± 0.8 dBi	8.8–10.5 dBi
**Size**	5.4λ × 5.4λ	1.8λ × 1.8λ	2.75λ × 2.75λ	2.75λ × 2.75λ	2.56λ × 2.56λ

## Data Availability

The data used in the paper can be available upon request to corresponding author.
